# Study of the absorption coefficient of graphene-polymer composites

**DOI:** 10.1038/s41598-018-27317-0

**Published:** 2018-06-14

**Authors:** K. Zeranska-Chudek, A. Lapinska, A. Wroblewska, J. Judek, A. Duzynska, M. Pawlowski, A. M. Witowski, M. Zdrojek

**Affiliations:** 1 Faculty of Physics, Warsaw University of Technology, Koszykowa 75, 00-662 Warsaw, Poland; 2 Faculty of Physics, Institute of Experimental Physics, University of Warsaw, Pasteura 5, 02-093 Warsaw, Poland

## Abstract

In this work, we have prepared a series of polydimethylsiloxane (PDMS) composites containing various graphene flakes loadings (0.02–2 wt%), and their broadband optical properties are being investigated. We demonstrate the tunability and evolution of transmittance and reflection spectra of the composites in a wide spectral range (0.4–200 μm) as a function of graphene content. Using these data we derive the broadband wavelength-dependent absorption coefficient (α) values. Our results show that α is roughly constant in the visible and IR ranges, and, surprisingly, is approximately one order of magnitude lower in the terahertz regime, suggesting different terahertz radiation scattering mechanism in our composite. Our material could be useful for applications in optical communication, sensing or ultrafast photonics.

## Introduction

Polymer composites combine flexibility and durability of an elastomer matrix with properties of the filler such as nanoparticles or graphene. The outstanding optical, electrical and mechanical properties of graphene flakes^1–3^ offer a possibility of potential application as a filler in synthetizing novel nanocomposites based on polymers^4–7^ that pave the way for future material applications (e.g. flexible electronics or photonics)^8^.

Literature provides numerous examples of graphene and other nanocarbon material based composites of different architecture and composition, prepared by a variety of methods^7,9–12^. Examples are as follows: a flexible and/or transparent graphene thin films^13–15^; graphene based 3D structures - graphene foam^16,17^ or hydrogel^18–20^ filled with polymer; multi-stacked sandwiches^21,22^; and other nanocarbon/polymer composites^23,24^. Remarkably, regardless of the production method, it has been shown that even a small loading of a nanocarbon filler can transfer its extraordinary properties into a composite material. For instance, mechanical and electrochemical reinforcement^25–28^ or boost of electrical and thermal transport properties^27,29,30^ that enable increased thermal conductivity^31–34^ and stability^27,35^, gases impermeability^36,37^, increase of electrochemical reactivity and sensing^38,39^, or efficient electromagnetic interference shielding^40,41^.

Carbon nanofillers such as graphene flakes offer a possibility of tuning the optical properties of the composite material. This is possible due to graphene’s particularly interesting optical features. It has been shown, for instance, that a single layer of graphene can absorb 2.3% of incident light^1^ in extremely broadband range. However, how this remarkable feature is transferred into graphene based composite material is still poorly studied and only several works have been written so far. For instance, graphene thin film (10 nm), composed of reduced graphene oxide sheets, deposited on polymer substrate exhibit a transmittance of 70%^42^. According to Bao *et al*.^43^ graphene load as small as 0.7 wt% in pristine polymer (PVAc) causes tenfold rise of the optical absorption of a composite in a UV-NIR range. Ultrathin graphene composite films can serve as an electrode for flexible electronics showing visible light transmittance in the range of 70–95% and can be tuned by graphene flake concentration^42,44^. Similar composites can be also applied as optical elements in fibre lasers, e.g. saturable absorbers^45,46^ or passive wave guides^47^.

In all above examples the tunability of optical properties is a key factor for progress in the application development. However, in literature the optical transmission/absorption of graphene based composites is investigated only in visible or near infrared range (still only few papers) and no systematic study on how the graphene content influence these properties can be found.

In this paper, we demonstrate a systematic study of broadband optical properties of graphene flakes immersed with variable concentration (0.02 to 2 wt%) in PDMS polymer matrix. We show the graphene-content dependent scalability of the absorption, transmission and reflectance properties of the composite in the wavelength range of 0.4–200 µm. For instance, our study shows that less than 0.5% of graphene content is enough to totally block the incoming radiation. Additionally, the experimental transmittance of our samples was compared with a model for stacked graphene multilayer. Finally, we derive broadband absorption coefficient (α) of the composite, that exhibit significantly different behaviour for VIS-IR and THz range, and its values strongly differ from other carbon material.

## Experimental

### Preparation of graphene/PDMS composite

The graphene-PDMS composites were prepared using conventional and simple blend mixing method. We used PDMS (Sylgard 184) supplied by Dow Corning Co. as a two-part liquid component kit (base and curing agent), since it is relatively transparent and has no colour, as compared to other commonly used polymer matrixes, i.e. poly methyl-vinyl siloxane rubber^48^ or styrene-butadiene rubber^49,50^. A specified amount of a few-layer graphene powder (Graphene Supermarket) with average thickness of several nanometers (4–10 ${\rm{nm}}$) and size of few micrometers (1.5–10 μm) was mixed with the base of PDMS solution. The mixture was then placed in a sonic bath for 2–3 hours (assisted by cooling system), followed by 1 hour mixing using a magnetic stirrer, and as a result a roughly homogenous distribution of graphene in PDMS base was obtained. Next, the 10:1 mixture (by weight, wt%) of PDMS base with graphene was mixed with curing agent followed by degassing step to remove air bubbles formed during the mixing process. The mixture was then cured at 100 °C for 1 h. Using the above procedure, series of ten samples were fabricated, containing different graphene concentration ranging from 0.02 to 2 wt%. In addition, a clean PDMS sample as a reference was also used. The thickness of all produced samples was approximately 800 µm (±10%).

**Figure 1:**
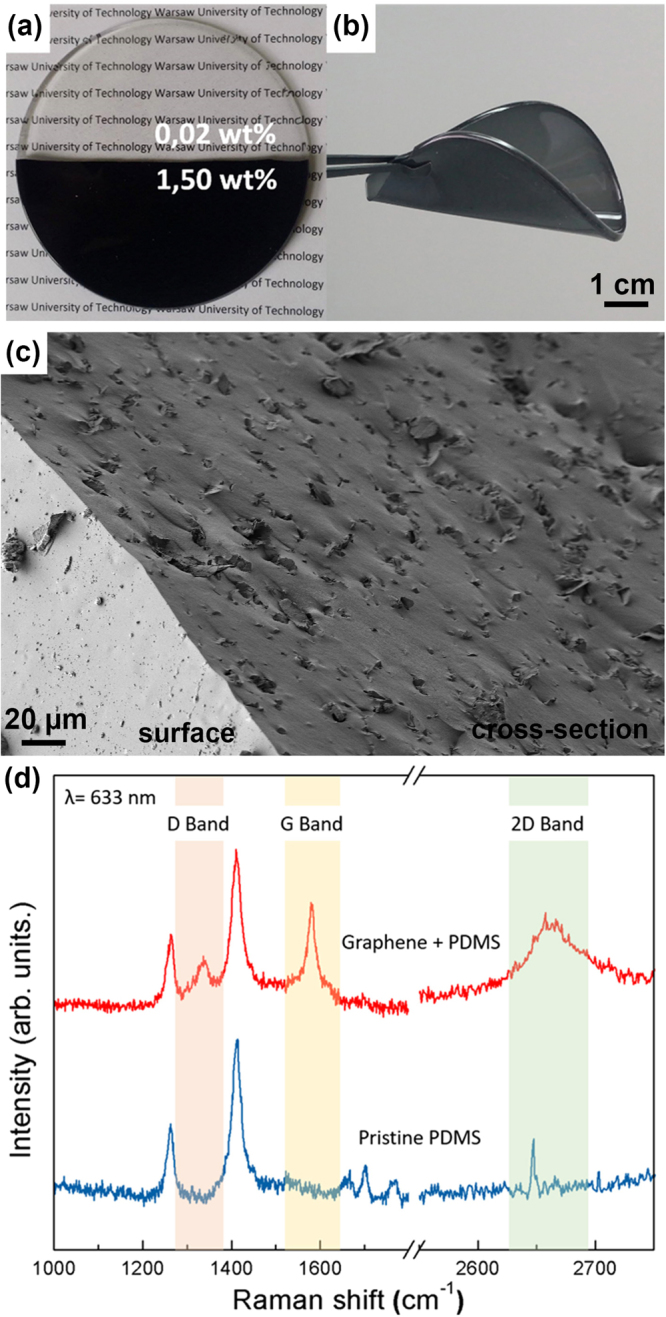
(**a**) Comparison of composites with different graphene loading. (**b**) Picture demonstrating the flexibility of produced composite. (**c**) SEM image of cross-section of the composite showing graphene flakes immersed in polymer matrix. (**d**) Raman spectra of pristine PDMS (lower curve) and graphene/PDMS composite (1 wt% graphene). The D, G and 2D bands characteristic for graphene material are highlighted.

The impact of the graphene loading level on the optical properties of the composite is already visible with the naked eye, as illustrated in Fig. 1(a), where samples with the lowest graphene content are almost fully transparent, whereas 1.5 wt% of graphene totally blocks visible light. Figure 1(b) demonstrates the flexibility of the composite owing to the elastomer matrix of PDMS^51^.

### Characterization of graphene/PDMS composites

To confirm the graphene presence and flakes deployment in the composite, we used Scanning Electron Microscope (Raith) and Raman spectroscope (Renishaw InVia). SEM image in Fig. 1(c) shows a cross-section of the composite with various flakes immersed in polymer matrix, which do not form a continuous network inside the material. Raman spectra where collected for reference, we measured a clean PDMS sample (no graphene) and a sample with 1 wt% graphene load (upper curve), as seen in Fig. 1(d). The highlighted bands: D (1348 cm^−1^), G (1588 cm^−1^) and 2D (2680 cm^−1^) are characteristic for graphene materials, thus are not observed in the pristine PDMS sample. The spectra did not show any changes for different graphene loading (except small changes in the intensity). The Raman experiment was conducted using 633 nm excitation wavelength (low power) and 50x objective, at room temperature.

The optical spectra were obtained using two different instruments depending on the wavelength range. For the VIS-NIR range (420–1700 nm) total reflectance (both specular and diffusive) and transmittance spectra were collected using a PV response analyser (PVE300 Bentham). For NIR-THz range (1.66–200 µm) only the transmittance measurements were conducted using a vacuum Fourier transform infrared spectrometer Bruker IFS-113v. The spectra were collected at the room temperature and ambient pressure (VIS-NIR range) or nitrogen pressure of about 6 mbr in the Bruker spectrometer. We then related obtained transmittance data to absorption coefficient $\alpha$ via equation (1)

1 $$T=\frac{{(1-R)}^{2}(1+{(\frac{\kappa }{n})}^{2})\exp (\,-\,\alpha d)}{1-{R}^{2}\exp (\,-\,2\alpha d)}$$

where *n* is the refractive index, *κ* is the extinction coefficient, T stands for transmittance, R is reflectance and $d$ is sample thickness. This relation can be simplified as $T={(1-R)}^{2}\exp (\,-\,\alpha d)$ and in cases of small reflectance as $T=\exp (\,-\,\alpha d)$. To relate the transmittance and absorption coefficient to the concentration of the nanofiller, one can use the Beer-Lambert law $T=\exp (\,-\,\alpha \cdot wt\cdot d)$, where $wt$ represents mass fraction of the carbon nanofiller. The absorption (A) spectra for VIS-NIR range were calculated using the following relation: $A=1-T-R$.

## Results and Discussion

**Figure 2:**
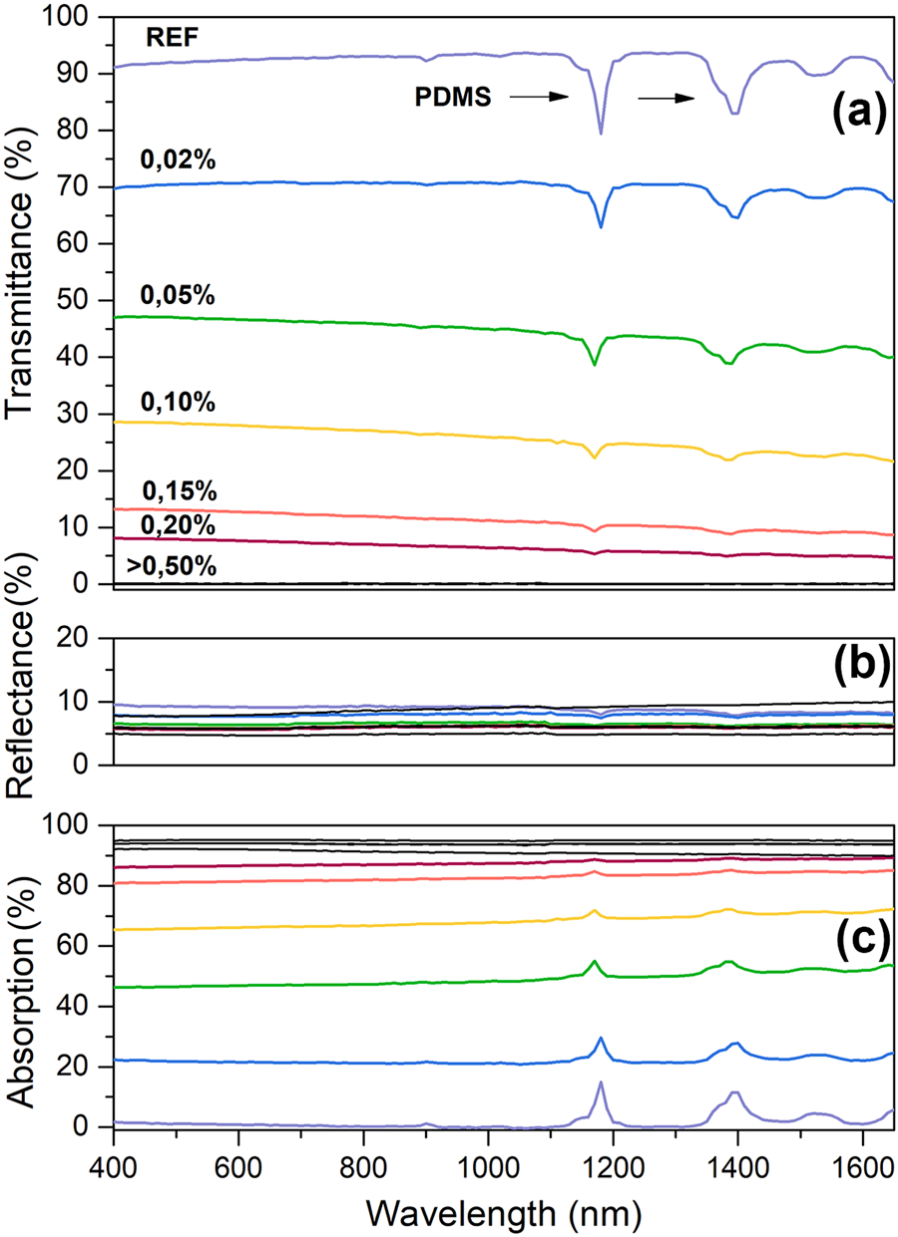
(**a**) Transmittance, (**b**) reflectance and (**c**) absorption spectra of graphene-PDMS composite samples (400 to 1700 nm range), together with PDMS characteristic drops marked with arrows. The % values correspond to graphene concentration, REF stands for pristine PDMS and 2% being the highest graphene concentration. The PDMS signatures disappear for samples with higher graphene loading due to increased absorption of the composite.

Figure 2(a) shows collected transmittance and reflectance spectra together with calculated absorption spectra in the VIS-NIR range for ten different graphene-PDMS composite samples.

All optical spectra remain relatively flat, except for small peaks around 1.15 µm, which are related to CH_3_ stretch vibrations characteristic for PDMS material^47^. Intuitively, an addition of given amount of graphene to the composite lowers the transparency almost evenly, without changing the shape of the spectra. This behaviour reflects the property of graphene monolayer which is constant within the optical range^1,24^. Thus, we show that our material exhibits a similar feature to mono- or multi-stacked graphene sandwich sample in the UV-IR range.

Clearly, the transmittance depends on the amount of graphene powder used in the process of fabrication. The more graphene is added to the composite, the less light is transmitted through the material. Interestingly, even a composite with the smallest graphene concentration (0.02 wt%) has significantly smaller transmittance (74% of incident light) as compared to the pristine PDMS sample (~94%). The transmittance level of a 0.02 wt% sample is comparable with a 10 nm thick layer of reduced graphene oxide on polymer reported by Wang *et al*.^42^, however our material is much easier to fabricate, requires no substrate. Moreover, it is easier for handling possible application.

The drop of the transmittance reaches a saturation point at 0.5 wt% graphene loading, where no light (VIS-NIR) is transmitted by the material, thus making it completely opaque in this range.

The optical reflectance level of all studied composites for VIS-NIR range, is kept in 4–8% range (see Fig. 2(b)). On the other hand, we note that pristine PDMS already has the reflectance level of ~7%^52^. For samples with higher graphene load the reflectance drops below this value, which might be related to a very high absorption level of those samples. This suggests that main contribution to the reflection comes from the polymer matrix, while graphene barely participate in the process or even cause the decrease of the reflection. The reflectance values of our composites are significantly lower than those of bulk graphite^53^ and approaching the values of mono- to a few layer graphene samples that show reflectance of roughly 1 to 2%^2^.

Both transmittance and reflectance data are then used to derive the absorption values of our composite for the VIS-NIR spectra range (see Fig. 2(c)). As only few percent of incident light is reflected off the material, the calculated absorption spectra is mostly determined by the transmittance and thus, it is directly related to graphene loading. The absorption as high as 95% is already exhibited by composites containing 0.5% (and more) graphene concentration. This suggests that blocking of light by graphene flakes dispersions can originate from two main mechanisms – absorption or multiple reflection (internal scattering)^54,55^. However, we note that due to the fact that the size of the flakes used in our material is roughly the same size as the wavelength of the incident light in the VIS-NIR spectral range and the non-zero value of absorption of the polymer matrix, radiation primarily scattered in the material would be absorbed before leaving the medium. Thus, it is likely that the mechanism based on internal multiple reflection is negligible. All this provides two conclusions: first, the main mechanism responsible for blocking the light in the studied range is the absorption and secondly, only small fraction of graphene in the material is needed to fully tune its optical properties.

**Figure 3:**
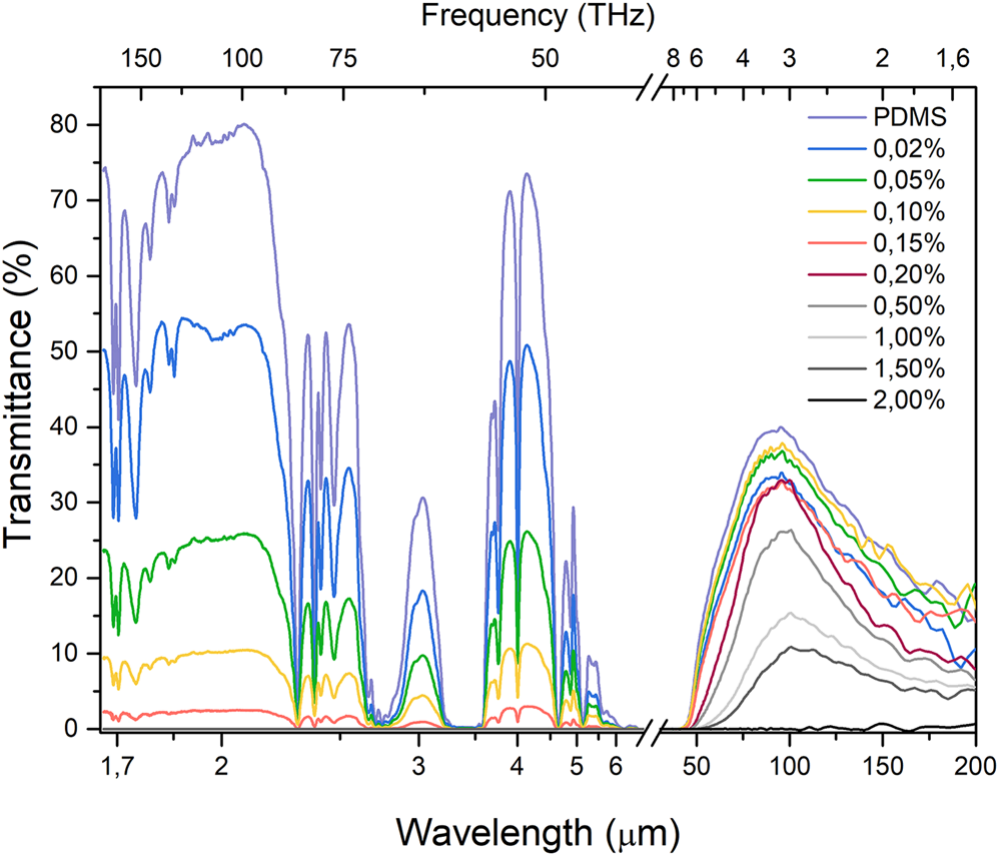
Transmittance spectra of graphene-PDMS composite in the wide spectral range (1400 nm–200 µm).

Next we discuss the transmittance spectra beyond VIS-NIR range, up to terahertz region (1.66–200 µm). Corresponding data are shown Fig. 3. Here, the spectra are strongly following the PDMS signatures^47^, while the values and their changes depend only on graphene loading. Although there are regions where PDMS transmittance signal is immeasurable, which may be due to relatively high thickness of the composite, we concentrate on “transmittance windows”, where the measurements of the influence of graphene flakes content is possible. In the IR region, again all composites with 0.5 wt% graphene concentration and higher show no measurable transmittance. Remarkably, this is not reproduced in the THz region, (for the wavelengths greater than 44 µm) our material is much more transparent with respect to the graphene content (e.g. sample with 1.5 wt% has ~10% transmittance at 3 T Hz).

In order to analyse the correlation between graphene loading and broadband transmittance of the fabricated composites in respect to the wavelength, the spectral range was divided into three parts – I: 400–1700 nm; II: 1, 66-6 μm and III: 44–200 μm. For each range a set of wavelengths was selected. Collected values of transmittance were then normalized according to the transmittance of pure PDMS, averaged within their ranges and presented in Table 1.

**Table 1:** Values of normalized transmittance (NT) in three ranges, in respect to the mass fraction and number of stacked graphene layers (N) for first two ranges.

graphene load (wt%)	Normalized Transmittance [%]			Corresponding number of graphene layers	
	spectral range				
	I 0.4–1.7 µm)	II (1.6–6 µm)	III (44–200 µm)	I	II
0.02%	78.5	63.7	87.0	10	19
0.05%	48.4	34.2	91.5	31	46
0.10%	27.3	15.5	89.0	56	80
0.15%	10.2	3.8	80.0	98	141
0.20%	5.7	2.9	77.3	123	152
0.50%	0.0	0.0	61.8	—	—
1.00%	0.0	0.0	35.9	—	—
1.50%	0.0	0.0	25.5	—	—
2.00%	0.0	0.0	0.0	—	—

It is interesting to compare the transmittance values of our composite with a simple model consisted of a number of graphene layers in a stack. In this case the transmittance of multilayer graphene is described by the following relation: $T(N)={(1-\pi {\alpha }_{fine})}^{N}$, where N is the number of layers and ${\alpha }_{fine}={e}^{2}/\hslash c$ is the fine structure constant^56^. We used this model to fit our data and match the obtained values of transmittance with corresponding number of graphene layer, in order to find a correlation between those two factors (see Table 1). Accordingly, for example material with 0.05 wt% graphene loading corresponds to a 30 layer (~10 nm thick) stack of graphene in terms of light transmission in the first spectral range, while in the IR region the same sample shows transmittance similar to approximately 50 graphene layers (~14 nm thick).

Although it is common knowledge that the transmittance of graphene sample depends on the number of stacked monolayers and is almost independent of the wavelength^1,2,57^ this feature is not preserved in a composite filled with graphene flakes. The data presented in Table 1 show major differences in the transmittance levels depending on the spectral range it was measured in. For instance, for a sample with the lowest graphene content the transmittance is ~78% in the first range and ~64% in the second range, which corresponds to 10 and 19 stacked graphene monolayers, respectively. These differences suggest different absorption mechanisms and as a result, different relation between graphene load and transmittance of the composite, depending on the wavelength of the incident radiation (see comments below).

**Figure 4:**
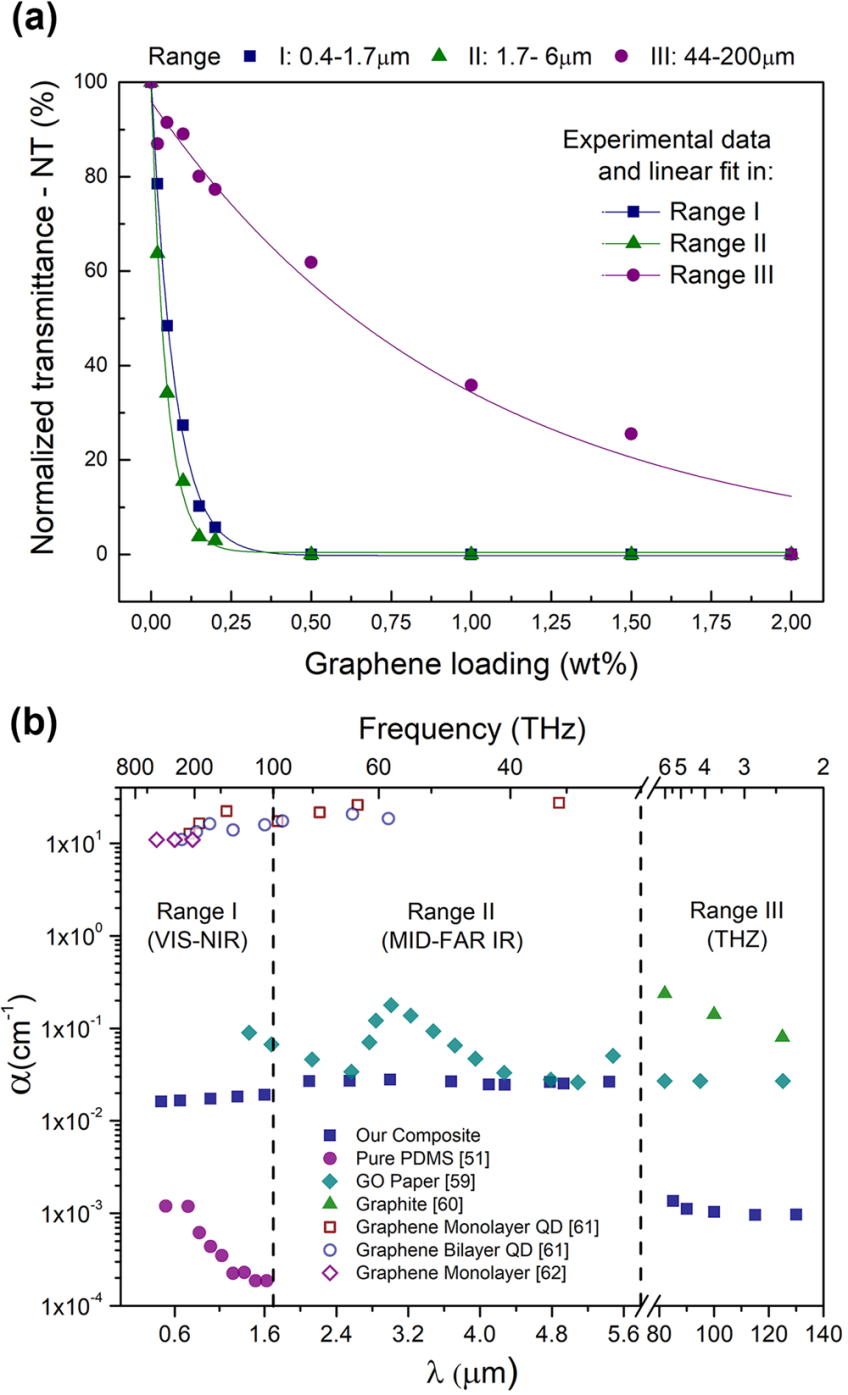
(**a**) Normalized transmittance as a function of graphene loading together with Beer-Lambert model (solid lines) plotted for three ranges. (**b**) Calculated absorption coefficient in respect to the wavelength (and frequency) in three spectral ranges compared with literature data for similar materials^51,59–62^.

The data from Table 1 are plotted in Fig. 4(a) and fitted with a modified model based on the Beer-Lambert law^58^ $T={A}_{R}\exp (-\alpha \cdot wt\cdot d)$, where $\alpha$ is the absorption coefficient, $wt$ is the concentration of graphene, $d$ represents the thickness of a composite and ${A}_{R}$ is a parameter compensating for different values of reflectance of pure PDMS and graphene/PDMS composite ($\sim 0.95$ in all ranges).

The trend of transmittance change as a function of graphene load is very similar in the first two ranges, where the transmittance values decrease substantially with the rising graphene concentration, up to 0.5 wt% graphene loading, when the material becomes non-transparent. Surprisingly, in the third range, the transmittance changes much differently (also exponential relation with different constant). The decrease slope of the transmittance is less steep as compared to first and second stages and values of T(wt%) reach zero only for the composite with 2 wt% load of graphene flakes. This demonstrates that the mechanisms behind the optical transmission of our composites exhibit significantly different behaviour for VIS-IR and THz range.

Those differences are even more apparent if we take into account the absorption coefficient ($\alpha )$ that can be derived using the aforementioned modified Beer-Lambert law. The values of absorption coefficient in respect to the wavelength are shown in Fig. 4(b) as filled squares and compared to other similar materials^51,59–62^. In the first and second range the $\alpha$ has similar values and can be averaged to a value of ${\alpha }_{I}\approx 170\,c{m}^{-1}$ and ${\alpha }_{II}\approx 270\,c{m}^{-1}$, respectively. In the third range values of the coefficient are significantly smaller ${\alpha }_{III}\approx 10\,c{m}^{-1}$, which is more than a decade lower with respect to VIS-IR range. Our results seem to imply that, when graphene is applied in a polymer composite, its transmittance is not independent of the wavelength. This effect likely stems from different attenuation mechanisms in the three spectral ranges. There are two major differences between the VIS-IR and THz ranges. Firstly, the wavelength in THz range is much longer than the average size of a graphene flake, which may strengthen the scattering mechanism of radiation in the composite. Secondly, although we don’t know the exact Fermi energy (${E}_{F}$) of graphene flakes, we can speculate that the energy of the signal is lower than $2{E}_{F}$, which weakens both scattering and absorption mechanisms. We argue that these two factors cause the absorption coefficient value to drop in Range III.

Calculated ${\alpha }_{I,II}$ can be compared with an absorption coefficient of graphene nanosheet dispersion determined by Coleman and Paton^62^, which is of the same order of magnitude or slightly higher. The exact value of the coefficient of graphene/polymer composite may vary due to the flake thickness and size distribution, as Su *et al*. report in their study on RGO (reduced graphene oxide) dispersion absorption coefficient^63^ and requires more thorough study.

Interestingly, it is striking that the values of ${\alpha }_{I,II}$ for graphene mono- and few layers^61,62^ are 3 orders of magnitude higher as compared to our materials, similarly if we compare ${\alpha }_{III}$ for graphite^49^ and our composite.

## Conclusions

Graphene flakes used as a nanofiller in a polymer composite have direct influence on the material transmittance. Filler concentration as small as 0.02 wt% affects the received material absorption, raising it 30 times compared to the pristine PDMS samples. That influence surpasses other works, which report absorption enhancement by 10 times with 0.07 wt% graphene loading^43^. It appears 0.5 wt% graphene concentration is sufficient to receive a material of almost 100% absorption of incoming radiation with the wavelength between 420 nm and 44 µm, while graphene load of 2 wt% creates a material that is nontransparent in the whole studied spectral range – up to 200 μm.

The influence graphene loading has on the transmittance of a composite can be compared with transmittance of stacked graphene multilayer. The 0.5 wt% of graphene flakes corresponds to a 90–150 nm thick multilayer graphene, but is much easier and more effective to produce in a form of a PDMS/graphene composite than through multiple delamination, or mechanical exfoliation, thus making the composite more desirable for possible applications.

Although the transmittance of pure graphene is known to be independent of wavelength, we show that when graphene is used as a nanofiller in a polymer composite, the transmittance changes as a function of the wavelength change due to different attenuation mechanisms. Calculated absorption coefficients also vary due to the wavelength. This change is most visible in the third spectral range (FIR), where absorption has significantly smaller value – ${\alpha }_{III} \sim \,10\,c{m}^{-1}$ in comparison to the first two ranges – ${\alpha }_{I,II} \sim 200\,\,c{m}^{-1}$.

### Data availability

The datasets generated and/or analysed during the current study are available from the corresponding author on reasonable request.
